# Overexpression of Pax6 results in microphthalmia, retinal dysplasia and defective retinal ganglion cell axon guidance

**DOI:** 10.1186/1471-213X-8-59

**Published:** 2008-05-28

**Authors:** Martine Manuel, Thomas Pratt, Min Liu, Glen Jeffery, David J Price

**Affiliations:** 1 Genes and Development Group, Centres for Integrative Physiology and Neuroscience Research, Hugh Robson Building, George Square, University of Edinburgh, Edinburgh EH8 9XD, UK; 2 Department of Visual Science, Institute of Ophthalmology, 11–43 Bath Street, London EC1V 9EL, UK

## Abstract

**Background:**

The transcription factor Pax6 is expressed by many cell types in the developing eye. Eyes do not form in homozygous loss-of-function mouse mutants (*Pax6*^*Sey*/*Sey*^) and are abnormally small in *Pax6*^*Sey*/+ ^mutants. Eyes are also abnormally small in *PAX77 *mice expressing multiple copies of human *PAX6 *in addition to endogenous *Pax6*; protein sequences are identical in the two species. The developmental events that lead to microphthalmia in *PAX77 *mice are not well-characterised, so it is not clear whether over- and under-expression of Pax6/PAX6 cause microphthalmia through similar mechanisms. Here, we examined the consequences of over-expression for the eye and its axonal connections.

**Results:**

Eyes form in *PAX77*^+/+ ^embryos but subsequently degenerate. At E12.5, we found no abnormalities in ocular morphology, retinal cell cycle parameters and the incidence of retinal cell death. From E14.5 on, we observed malformations of the optic disc. From E16.5 into postnatal life there is progressively more severe retinal dysplasia and microphthalmia. Analyses of patterns of gene expression indicated that *PAX77*^+/+ ^retinae produce a normal range of cell types, including retinal ganglion cells (RGCs). At E14.5 and E16.5, quantitative RT-PCR with probes for a range of molecules associated with retinal development showed only one significant change: a slight reduction in levels of mRNA encoding the secreted morphogen *Shh *at E16.5. At E16.5, tract-tracing with carbocyanine dyes in *PAX77*^+/+ ^embryos revealed errors in intraretinal navigation by RGC axons, a decrease in the number of RGC axons reaching the thalamus and an increase in the proportion of ipsilateral projections among those RGC axons that do reach the thalamus. A survey of embryos with different *Pax6/PAX6 *gene dosage (*Pax6*^*Sey*/+^, *Pax6*^+/+^, *PAX77*^+ ^and *PAX77*^+/+^) showed that (1) the total number of RGC axons projected by the retina and (2) the proportions that are sorted into the ipsilateral and contralateral optic tracts at the optic chiasm vary differently with gene dosage. Increasing dosage increases the proportion projecting ipsilaterally regardless of the size of the total projection.

**Conclusion:**

Pax6 overexpression does not obviously impair the initial formation of the eye and its major cell-types but prevents normal development of the retina from about E14.5, leading eventually to severe retinal degeneration in postnatal life. This sequence is different to that underlying microphthalmia in *Pax6*^+/- ^heterozygotes, which is due primarily to defects in the initial stages of lens formation. Before the onset of severe retinal dysplasia, Pax6 overexpression causes defects of retinal axons, preventing their normal growth and navigation through the optic chiasm.

## Background

The transcription factor Pax6 is expressed dynamically during the early development of the mouse eye in both the surface ectoderm and the optic vesicle, which integrate to generate the structures of the eye [1-3]. As morphogenesis proceeds, Pax6 is expressed in the lens, retinal pigment epithelium and retina [1-4]. Most studies examining the functions of Pax6 in eye development have exploited loss-of-function models. The optic cup fails to form in embryos completely lacking Pax6 [5]. Examination of *Pax6*^+/+ ^↔ *Pax6*^-/- ^mouse chimeras has shown that Pax6 is required cell autonomously in the optic vesicle for maintenance of contact with the overlying surface ectoderm, a necessary event in eye formation, providing a clue that Pax6 may be involved in defining the adhesive properties of these cells [6,7]. Heterozygous *Pax6*^+/- ^embryos show microphthalmia that is thought to be caused by delayed development of the lens and anterior eye structures [8]. Data from *Pax6*^+/- ^↔ *Pax6*^+/- ^chimeras has demonstrated that heterozygous cells have early cell autonomous deficiencies at lens placode formation and after closure of the lens pit [9]. Studies using Cre-lox technology to selectively disrupt *Pax6 *in discrete parts of the developing eye have shown that removing Pax6 from the developing surface ectoderm produces an eye lacking a lens but possessing a retina with retinal ganglion cells (RGCs) able to project axons [10]. Removing Pax6 function after the retina forms results in a retina comprising mainly amacrine cells at the expense of other retinal cell types including RGCs [3].

Gain-of-function models are also useful for assessing gene function, particularly where, as in the case of Pax6, absence of function leads to complete abolition of a structure or cell type. In some parts of the developing brain Pax6 has functions in axon guidance as well as in tissue morphogenesis and regulates genes implicated in growth cone navigation [11]. Since absence of Pax6 results in absence of RGCs, it is unclear whether Pax6 has a role in the navigation of retinal axons. Its expression by projecting RGCs [4] indicates that it is poised to fulfil this function. Here, we investigated the formation of the eye, retina and retinal projections in mice that over-express Pax6 and we complemented our analysis of retinal projections in the gain-of-function model by studying retinal projections in *Pax6*^+/- ^embryos.

Controlled overexpression of Pax6 in its normal domains of expression has been achieved by the generation of a YAC transgenic mouse expressing several copies of the human *PAX6 *gene, which generates protein identical to mouse Pax6, under the control of all its regulatory elements [12]. PAX77 hemizygous mice carry 5 to 7 copies of the human *PAX6 *gene, all integrated at the same locus, and are designated *PAX77*^+ ^[12]. PAX77 homozygous transgenic mice are designated *PAX77*^+/+^. Schedl et al. [12] showed that over-expression of PAX6 causes microphthalmia in adults but did not study the development of this defect. Here we show that the eye initially forms apparently normally but that retinal defects and microphthalmia emerge and become increasingly severe during subsequent embryogenesis and postnatal life. We also show that Pax6 overexpression causes defects in RGC axon growth and guidance through the optic chiasm, where RGC axons are sorted into those that project ipsilaterally and those that project contralaterally. Our results indicate that although both over- and under-expression of Pax6 cause microphthalmia, the underlying mechanisms are different.

## Results

### Pax6/PAX6 protein and mRNA levels are increased in the eye of PAX77 embryos

**Figure 1:**
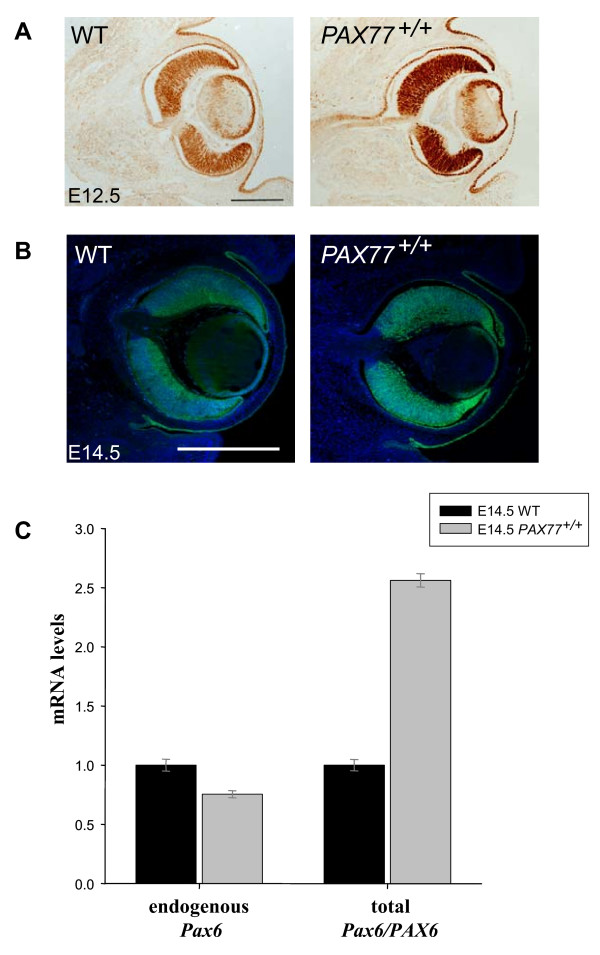
**Pax6 is overexpressed in the eye of *PAX77*^+/+ ^embryos.** Horizontal sections through the eye of wild-type and *PAX77*^+/+ ^embryos at (A) E12.5 and (B) E14.5, showing the expression of Pax6/PAX6. (C) Levels of endogenous *Pax6 *mRNA and total *Pax6/PAX6 *mRNA in the retina of E14.5 *PAX77*^+/+ ^embryos, relative to wild-types, determined by real time quantitative RT-PCR and normalised against GAPDH mRNA levels (n = 3 in each case).*Pax6 *mRNA levels are significantly decreased in the retinae of *PAX77*^+/+ ^embryos compared to those of wild-types (Student's t-test, p = 0.014) while *Pax6/PAX6 *mRNA levels are significantly increased (Student's t-test, p < 0.001). Scale bars: A, 200 μm; B, 500 μm.

We compared Pax6/PAX6 (mouse + human) expression in wild-type and *PAX77*^+/+ ^eyes by immunohistochemistry on sections of E12.5 and E14.5 embryos. The pattern of expression of Pax6/PAX6 in the eye of *PAX77*^+/+ ^embryos reproduces that in the wild-types (Fig 1A, B). As expected, the levels of Pax6/PAX6 in *PAX77*^+/+ ^eyes appear increased compared to those in wild-types (Fig 1A, B). We also measured the levels of specifically mouse *Pax6 *mRNA and both mouse and human *Pax6*/*PAX6 *mRNA in the retinae of E14.5 wild-type and *PAX77*^+/+ ^embryos by quantitative RT-PCR (qRT-PCR). We found that the total levels of *Pax6/PAX6 *mRNA are increased by about 2.5 fold in *PAX77*^+/+ ^compared to wild-type retinae (Fig 1C). This increase is lower than might be expected from the increase in gene copy number (5–7 fold), suggesting that Pax6 negatively autoregulates in the retina, as it does in the brain [13]. In agreement with this, we found that the levels of endogenous mouse *Pax6 *mRNA are decreased in *PAX77*^+/+ ^retinae compared to wild-type retinae (Fig 1C), indicating that overproduction of PAX6 from the YAC transgenes represses production from the endogenous *Pax6 *loci. For convenience, we refer elsewhere in this paper to the overexpression in PAX77 mice as being an overproduction of Pax6, rather than Pax6/PAX6, since the human and mouse protein sequences are identical.

### Eye development until E12.5 is similar in wild-type and Pax6-overexpressing embryos

**Figure 2:**
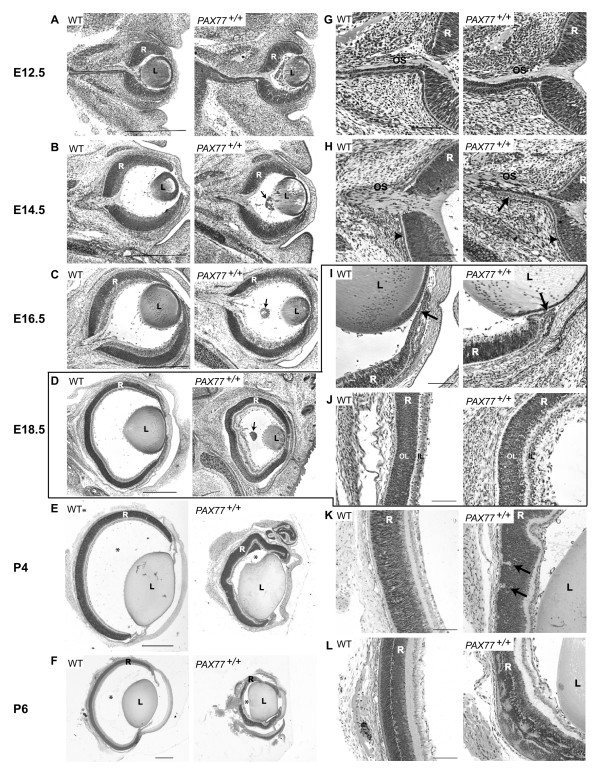
**Overexpression of Pax6 in the eye results in microphthalmia and retinal dysplasia.** Horizontal sections through the eyes of wild-type and *PAX77*^+/+ ^eyes stained with cresyl violet. (A, G) E12.5, (B,H) E14.5, (C) E16.5, (D,I,J) E18.5, (E,K) P4, (F,L) P6. (A-F) As the eye develops the retina of *PAX77*^+/+ ^mice becomes progressively smaller than in wild-type mice. (B,C,D) A retrolental nodule (arrow) is present in the mutant eye from E14.5. (E,F) After birth, the vitreous chamber (*) has almost completely disappeared in the *PAX77*^+/+ ^eye and parts of the retina are in close contact with the lens. (H) Cells continuous with the pigmented epithelium (arrowhead) and the retina extend ectopically along the mutant optic stalk (arrow). (I) At E18.5, in the *PAX77*^+/+ ^eye the iris (arrow) is folded and in direct contact with the lens epithelium. (J) At E18.5 the retina is comprised of 2 well defined neuroblastic layers in mutant as in wild-type embryos. (K) At P4 the outer layer of the *PAX77*^+/+ ^retina starts to form rosettes (arrows). (L) At P6 *PAX77*^+/+ ^eyes display variable levels of rosetting of the outer nuclear layer. The *PAX77*^+/+ ^eye shown here displays severe rosetting. L, lens; OS, optic stalk; R, retina; OL, outer layer; IL, inner layer. Scale bars : A-F, 500 μm; G-L, 100 μm.

**Figure 3:**
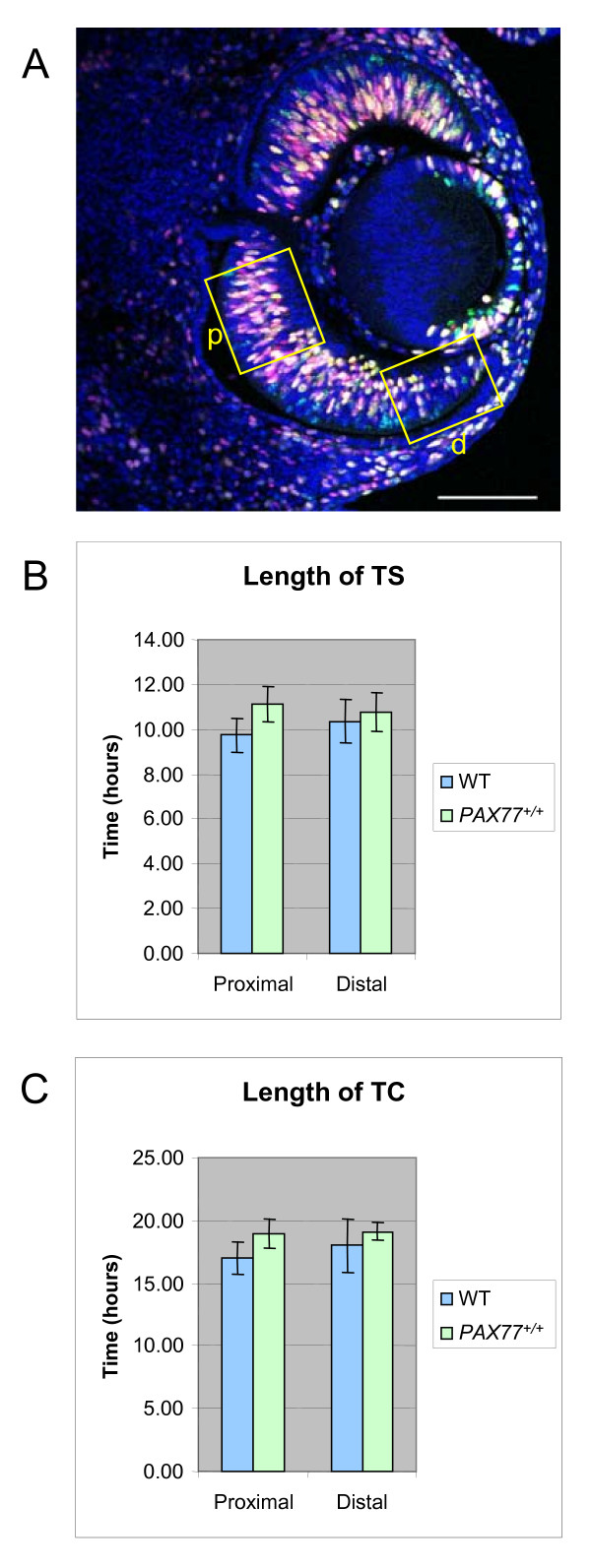
***Pax6* overexpression does not significantly affect retinal progenitor proliferation at E12.5 (A) Horizontal section of the eye of an E12.5 wild-type embryo labeled with anti-BrdU (red), anti-IdU (green) and counterstained with TOPRO3 (blue).** Cell counts were made in 100 μm wide sampling boxes in the proximal (p) and distal (d) retina. (B) Estimated S-phase length (TS) and (C) estimated cell cycle length (TC) in the proximal and distal retina of wild-type and *PAX77*^+/+ ^E12.5 embryos. No significant differences were detected between the mutants and the wild-types (n = 3 of each; Student's t-test; p > 0.05). Scale bar: 100 μm.

At early stages of embryogenesis, up to E12.5, the eyes of *PAX77*^+/+ ^embryos appear similar to those of the wild-types in both size and morphology (Fig 2A, G). They do not show the lens defects reported in *Pax6*^+/- ^embryos at this age [8,9]. We tested for more subtle defects of cell proliferation and death at this age but found no evidence for either. We used the technique of sequential labeling of cells in S-phase with BrdU and IdU [14,15] to estimate the cell-cycle length (TC) and the length of the S-phase (TS) of retinal progenitors (Fig 3). We did not find any significant difference in the estimated TS (Fig 3B) or TC (Fig 3C) in the proximal and distal retina of E12.5 *PAX77*^+/+ ^embryos compared to wild-types (P > 0.05, Student's t test, n = 3 wild-type and 3 *PAX77*^+/+ ^embryos). We performed an analysis of cell death in the retina by examining the pattern of dying cells clearly visible on thin plastic sections as pyknotic nuclei. Pyknosis is the most characteristic feature of apoptosis and counting pyknotic nuclei is a standard way of looking at cell death in the retina [16-30]. In a previous study we counted apoptotic cells in the brain by counting pyknotic nuclei as well as by counting Terminal Uridine Deoxynucleotidyl Transferase dUTP Nick End Labeling (TUNEL) positive cells and found that both techniques gave the same result [31].We did not observe any obvious increase in the number of pyknotic figures per 2 μm section through the eye of E12.5 *PAX77*^+/+ ^embryos compared to wild-types. In sections from eyes of both genotypes there were between 0 and 2 pyknotic figures per section. Thus, there does not appear to be a dysregulation of progenitor proliferation and death at early stages of retinogenesis in *PAX77*^+/+ ^embryos.

### Overexpression of Pax6 results in optic disc malformations, progressive retinal dysplasia and microphthalmia from E14.5

As development progresses *PAX77*^+/+ ^retinae become progressively smaller than normal while the size and appearance of their lenses appears relatively less affected (Fig 2B–F). As a consequence, from postnatal day (P) 2 their vitreous chambers have almost completely disappeared and parts of their retinae and lenses are in direct contact (Fig 2E, F).

Morphological defects are first detected at E14.5 with the development of persistent hyperplastic primary vitreous (PHPV), a condition that arises when the hyaloid canal does not retract appropriately towards the optic nerve head leaving a tissue nodule (arrow in Fig 2B–D) [32], and malformations of the optic disc (Fig 2B, H). At this stage in the wild-type, the pigmented epithelium (arrowhead in Fig. 2H) and the retinal neuroepithelium stop sharply at the junction of the optic cup and optic stalk, whereas in the mutant, cells continuous with those of pigmented epithelium and neural retinal cells extend along the optic stalk (arrow in Fig 2H). Taken together, these results indicate that failure of the optic nerve head to develop normally is an early feature in *PAX77*^+/+ ^embryos. Defects of RGC axons at around this time are analysed in greater detail below.

**Figure 4:**
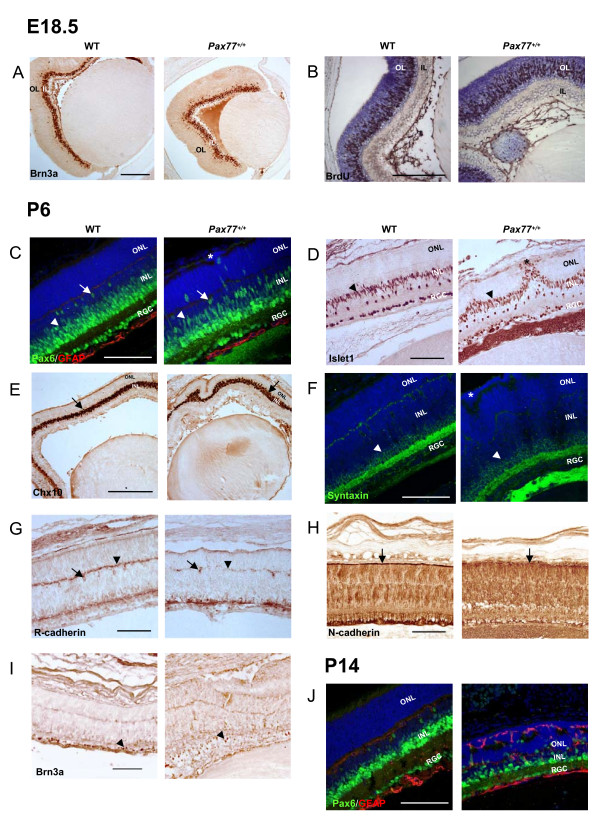
**The different retinal cell types are produced normally in the *PAX77*^+/+ ^eye.** Horizontal sections through wild-type and *PAX77*^+/+ ^eyes. (A) Expression of the RGC marker Brn3a at E18.5. (B) BrdU labelling (brown) of retinal progenitors in S-phase in E18.5 eyes. Sections are counterstained with cresyl violet. Expression of (C, J) Pax6 (green) and GFAP (red), (D) Islet1, (E) Chx10, (F) Syntaxin, (G) R-cadherin, (H) N-cadherin and (I) Brn3a in the retinae of wild-type and *PAX77*^+/+ ^pups at P6 (C to I) and P14 (J). (C,F) Sections are counterstained with TOPRO3. Arrowheads in C,D,F: amacrine cells; arrows in C,G: horizontal cells; arrows in E: bipolar and progenitor cells; arrowhead in G: outer plexiform layer; arrow in H: outer limiting membrane; stars in C,D,F: ectopic retinal cells; arrowheads in I: RGCs. OL, outer layer; IL, inner layer; ONL, outer nuclear layer; INL inner nuclear layer; RGC, retinal ganglion cells. Scale bars : A, 250 μm; B, 150 μm; E, 300 μm; C,D,F,G,H,I,J, 100 μm.

By E18.5 the retinae of *PAX77*^+/+ ^eyes have become significantly smaller than those of the wild-type eyes (Fig 2D) and the iris appears folded and pushed against the lens epithelium (arrow in Fig 2I). At E18.5, the wild-type retina comprises two well-defined layers. The inner layer is the differentiated RGC layer and the outer layer is the region of the retina that is still primarily neuroblastic. While both layers are present in the mutant retina, the RGC layer appears to be less structured than in the wild-type retina (Fig. 2J). Four days after birth, when normal neuronal production is declining rapidly and the retina is initiating the differentiation of the inner and outer nuclear layers, rosette-like structures are starting to form in the outer layer of the retina of *PAX77*^+/+ ^mice (Fig 2E, K). In addition, the peripheral retina is folded back towards what remains of the vitreous chamber and retinal folds appear more centrally (Fig 2E). At P6, *PAX77*^+/+ ^retinae display degrees of rosetting that vary between eyes and also between different regions of the same eye. Figures 2F and 2L show a *PAX77*^+/+ ^eye at P6 with very severe rosetting. In all P14 *PAX77*^+/+ ^eyes examined the entire outer nuclear layer consisted of dysplastic rosettes (Fig 4J) and the retina displayed extensive gliosis (shown by glial fibrillary acidic protein [GFAP] staining in Fig 4J).

**Figure 5:**
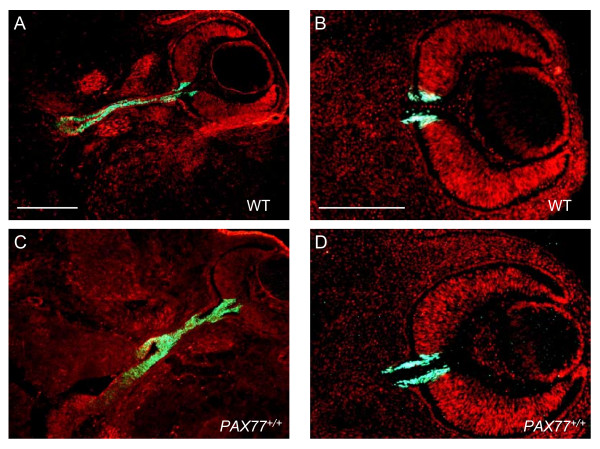
**Pax2 appears to be expressed normally in the optic stalk of the *PAX77*^+/+ ^eye.** Horizontal sections through the eye of (A,B) wild-type and (C,D) *PAX77*^+/+ ^E12.5 embryos showing the expression of Pax2 in the optic stalk. Sections are counterstained with TOPRO3. Scale bars: A,C, 300 μm; B,D, 200 μm.

The abnormality of the optic disc observed in the mutant embryos from E14.5 is similar to that previously described in mice heterozygous for the Kidney and retinal defects (krd) deletion, which display haploinsufficiency of the transcription factor Pax2 [33]. We could not, however, detect by immunofluorescence any obvious difference in the pattern or the level of expression of Pax2 in the eyes of *PAX77*^+/+ ^embryos compared to wild-types (Fig 5).

### Analysis of patterns of gene expression in the retinae of PAX77^+/+ ^and wild-type mice

In the retina of wild-types aged E18.5, proliferative cells are found in the outer layer while the inner layer contains the recently differentiated RGCs. We analyzed the organization of the retina at E18.5 using Brn3a (Fig 4A) as a marker of RGCs [34,35] and BrdU to label proliferative cells in S-phase (Fig 4B). There are no obvious differences in the positions and densities of cells labelled with BrdU or Brn3a between *PAX77*^+/+ ^and wild-type retina (Fig 4A, B). A further quantitative analysis of Brn3a labelling in wild-type and mutant embryos, presented below in the context of RGC axonal development, supported these findings. Thus, at E18.5, although the *PAX77*^+/+ ^retina is smaller than the wild-type retina, its organization appears to be normal.

We analyzed the expression of a broader range of markers in the postnatal retina, at P6. The RGCs, which express Pax6, Islet1 and Brn3a [3,36], are present in the mutant retina but they are not as regularly aligned as in the wild-type retina (Fig 4C, D and arrowheads in Fig 4I). Pax6, Islet1 and Syntaxin are expressed by amacrine cells [3,36] (Fig 4C, D, F; arrowheads), Chx10 is a marker of bipolar and retinal progenitor cells [37] (Fig 4E; arrows) and Pax6 and R-cadherin are expressed by horizontal cells [3,36,38] (Fig 4C, G; arrows). Analysis of the expression of these markers in *PAX77*^+/+ ^retina provided evidence that all the major cell types of the inner nuclear layer are produced in the mutant, but their appearances and distributions are not normal at P6. Cells expressing markers associated with cells from the inner nuclear layer accumulate at the outer surface of the retina (Fig 4C, D, F; stars); their appearance suggests that they might have migrated through the outer nuclear layer. In addition, there seem to be fewer horizontal cells in the *PAX77*^+/+ ^retina than in the wild-type retina and they are not as regularly spaced (Fig 4C, G; arrows). In the wild-type retina, R-cadherin is strongly expressed in the outer plexiform layer, which contains the horizontal cells' dendrites (Fig 4G; arrowheads). The expression of R-cadherin in the outer plexiform layer of the mutant retina is much weaker and does not appear as a continuous line as in the wild-type (Fig 4G). This suggests that the horizontal cells in the mutant retina do not form normal dendritic connections with cells in the outer nuclear layer. In the wild-type retina, the outer limiting membrane is strongly stained by antibodies against N-cadherin [38,39] (Fig 4H; arrow). The outer limiting membrane in the *PAX77*^+/+ ^eye is not as clearly defined as in the wild-type and it appears discontinuous (Fig 4H; arrow). Overall, these results indicate that the major cell types of the normal retina are produced in the mutants but they are disorganized at P6.

We used real time quantitative RT-PCR to analyze the mRNA levels of several genes which are implicated in retina formation and RGC axon navigation and some of which are known targets of regulation by Pax6. We compared the mRNA levels of our candidates in the retinae of wild-type and *PAX77*^+/+ ^embryos at E14.5 and E16.5, stages at which retinal dysmorphology first emerges in the mutants (Table 1). Shh, which is expressed specifically by the RGCs [40,41], is implicated in retinal lamination and RGC axon guidance [40,41]. We found a small, significant decrease in the levels of Shh mRNA in the *PAX77*^+/+ ^retinae compared to the wild-types at E16.5 (Table 1). We also analyzed the expression of the adhesion molecules Ncam, R-cadherin and L1 which are implicated in axon growth and guidance and are transcriptional targets of Pax6 [11,42-45]. L1, in particular, is essential for topographic mapping of retinal axons [43]. We did not detect any difference in expression of these genes in the *PAX77*^+/+ ^retina compared to the wild-type. The homeodomain transcription factor Six3 is an important regulator of retinal development and may regulate retinal progenitor proliferation and cell fate specification [46-48]. There is evidence that the expression of Six3 is regulated by Pax6 [49]. The bHLH transcription factor Ngn2 has been implicated in the regulation of the specification of RGCs [50] and is a transcriptional target of Pax6 in the telencephalon and spinal chord [51,52]. The expression of Six3 and Ngn2 was not significantly altered in the *PAX77*^+/+ ^compared to the wild-type retina.

**Table 1:** Relative gene expression in *PAX77*^+/+ ^and wild-type embryonic retinae

		**ratio *PAX77*^+/+^/WT**	
		
**Shh**	**E14.5**	0.997	p = 0.982
	**E16.5**	0.839	p = 0.006 *
**Ncam**	**E14.5**	1.040	p = 0.488
	**E16.5**	0.933	p = 0.356
**Rcad**	**E14.5**	0.827	p = 0.351
	**E16.5**	1.044	p = 0.744
**Six3**	**E14.5**	0.963	p = 0.667
	**E16.5**	0.937	p = 0.474
**L1**	**E14.5**	1.180	p = 0.365
	**E16.5**	1.135	p = 0.239
**Ngn2**	**E14.5**	0.878	p = 0.342
	**E16.5**	0.907	p = 0.425

Shh mRNA levels are slightly decreased in the retinae of E16.5 *PAX77*^+/+ ^embryos compared to the wild-types. mRNA levels of potential targets of Pax6 in the retina of E14.5 or E16.5 *PAX77*^+/+ ^embryos, relative to wild-type, determined by real time quantitative RT-PCR and normalised against GAPDH mRNA levels (n = 3 to 6). P values were given by Student's t-test.

### Overexpression of Pax6 results in intraretinal and retinothalamic errors in RGC axon navigation

**Figure 6:**
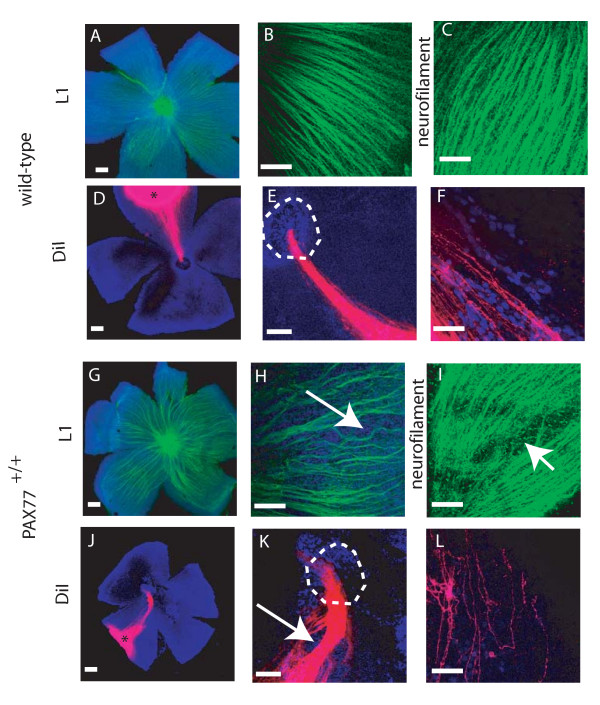
**Alterations to RGC axon trajectories within the *PAX77*^+/+ ^retina at E16.5.** (A-F) Wild-type (G-L) *PAX77*^+/+^: panels show flatmounted retinae in which retinal axons are detected using (A,B,G.H) L1 immunostaining, (C,I) neurofilament immunostaining, or (D-F, J-L) DiI labelling following focal injection of DiI into the peripheral retina. In wild-type embryos retinal axons grow directly towards the optic nerve head in the centre of the retina. In *PAX77*^+/+ ^embryos, although this radial organisation is grossly preserved, the retinal axons take a more erratic route. (B,C) In the wild-type axon bundles are uniformly spaced and appear parallel when viewed at high magnification. (D,E) DiI focal injections label cohorts of axons which grow straight towards the optic nerve head (demarcated by dotted circle in E,K). In contrast in *PAX77*^+/+ ^embryos axon bundles are less evenly distributed and avoid some parts of the retina altogether (arrows in H,I,K). (F,L) Higher magnification of DiI labelled axons in the retina showing individual axons. (F) In the wild-type these run parallel to one another but (L) in the *PAX77*^+/+ ^retina they do not. Scale bars: (A,D,E,G,J,K), 100 μm; (B,C,F,H,I,L), 50 μm.

In order to test the hypothesis that normal levels of Pax6 are important for the proper navigation of RGC axons we first examined the ability of RGC axons to navigate within the *PAX77*^+/+ ^retina. We looked at E16.5 retinae as, by this age in wild-types, large numbers of RGC axons have grown to the optic nerve head and any defects in intraretinal navigation in mutants would be apparent. In wild-type embryos, RGC axons grow radially towards the optic nerve head as revealed by immunostaining RGC axons for L1 (Fig. 6A, B) or neurofilament (Fig. 6C), or labelling cohorts of RGC axons by DiI injection into the peripheral retina (Fig. 6D–F). In contrast, in *PAX77*^+/+ ^embryos RGC axons take a more erratic path. L1 (Fig. 6G, H) and neurofilament (Fig. 6I) immunostaining showed that, as in the wild-type, RGC axons form bundles that converge on the optic nerve head. However, whereas in the wild-type these bundles run approximately linearly and cover the retinal surface evenly, in the *PAX77*^+/+ ^embryo they take a more serpentine course and are not evenly distributed across the retinal surface. Focal DiI injections labelling cohorts of RGC axons (Fig. 6J–L) showed that although many axons do converge on the optic nerve head their course is erratic.

**Figure 7:**
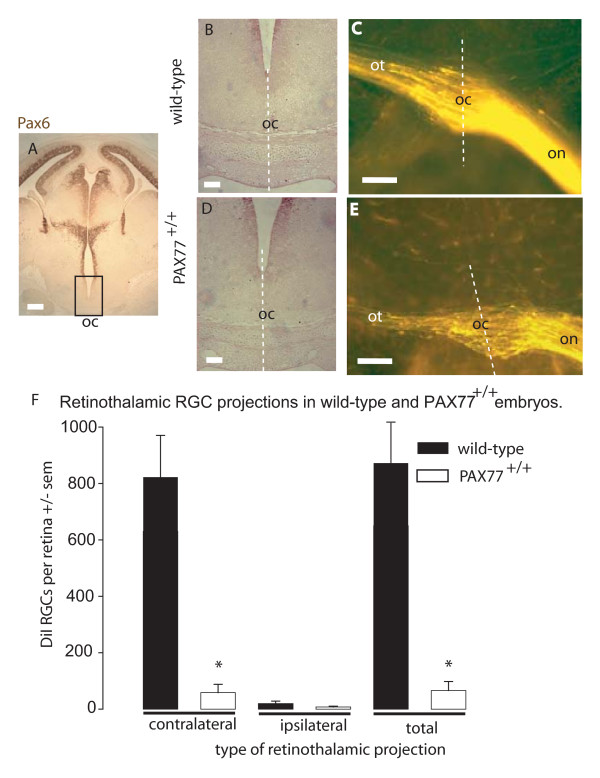
**Alterations to RGC axon numbers and behaviour at the optic chiasm of *PAX77*^+/+ ^embryos.** (A,B,D) Pax6 immunostaining of E14.5 coronal section. (A) In the wild-type Pax6 expression is not detectable at the optic chiasm (oc) although it is evident in other parts of the brain. Higher magnification of the oc of (B) wild-type and (D) *PAX77*^+/+ ^embryos show that in neither case is Pax6 immunostaining detectable. (C, E) Horizontal sections showing DiI-labelled retinal axons as they traverse the chiasm, following injections of label into the retina. In both (C) wild-type and (E) *PAX77*^+/+ ^embryos, retinal axons reach the chiasm along the optic nerve (on) and follow similar trajectories into the optic tract (ot). White dotted lines in (B-E) demarcate the midline. (F) Quantification of RGC projections to the thalamus in wild-type and *PAX77*^+/+ ^embryos at E16.5 obtained by injecting DiI into the thalamus (on one side only) and counting RGCs retrogradely labelled by DiI in the ipsilateral and contralateral retinae. Note that *PAX77*^+/+ ^embryos exhibit a dramatic decrease in the number of contralaterally projecting RGCs which is statistically significant (p = 0.008; Mann-Whitney Rank Sum Test). The slight reduction in the number of ipsilaterally projecting RGCs is not significant (p = 0.317; Rank Sum Test). * indicates significant difference (p < 0.05; Mann-Whitney Rank Sum Test). Number of embryos analysed: *Pax6*^+/+ ^n = 9; *PAX77*^+/+ ^n = 4. Scale bars: (A) 500 μm; (B-E) 100 μm.

Despite exhibiting abnormal routing within the retina, a significant number of RGC axons exit the retina at the optic nerve head and grow into the optic stalk in *PAX77*^+/+ ^embryos. During normal development the optic nerves converge on the ventral surface of the hypothalamus to form the optic chiasm. Axons are then sorted into the contralateral or ipsilateral optic tracts and navigate to their targets in the thalamus and superior colliculus. Immunostaining for Pax6 in wild-type (Fig. 7A, B) and *PAX77*^+/+ ^(Fig. 7D) embryos shows that Pax6 is not expressed at detectable levels at the optic chiasm at E14.5, the age at which RGC axons are navigating this region. Horizontal sections of wild-type (Fig. 7C) and *PAX77*^+/+ ^(Fig. 7E) optic chiasm following DiI injection into the retina reveal that in both cases significant numbers of axons reach the chiasm and continue into the optic tracts. We noticed that our injections consistently labelled more axons in the wild-type than in the *PAX77*^+/+ ^embryos suggesting that fewer axons reached this region in the *PAX77*^+/+ ^embryos. In order to quantify this we counted the numbers of RGC cell bodies retrogradely labelled by unilateral DiI injection into the thalamus at E16.5 (Fig. 7F). These counts confirmed our initial observation that the *PAX77*^+/+ ^retina projects fewer axons to the thalamus: the total number of back-labelled RGCs in the *PAX77*^+/+ ^retina was reduced compared to the wild-type (p < 0.05; Mann-Whitney Rank Sum Test). Interestingly, however, there was a disproportionate reduction in the contralateral projection which was reduced by 90% in the *PAX77*^+/+ ^embryos (p < 0.05; Mann-Whitney Rank Sum Test) compared to the ipsilateral projection in which there was no significant decrease (p > 0.05; Mann-Whitney Rank Sum Test). This is examined in more detail below.

### The number of RGCs in the retina is sensitive to Pax6 gene dosage

**Figure 8:**
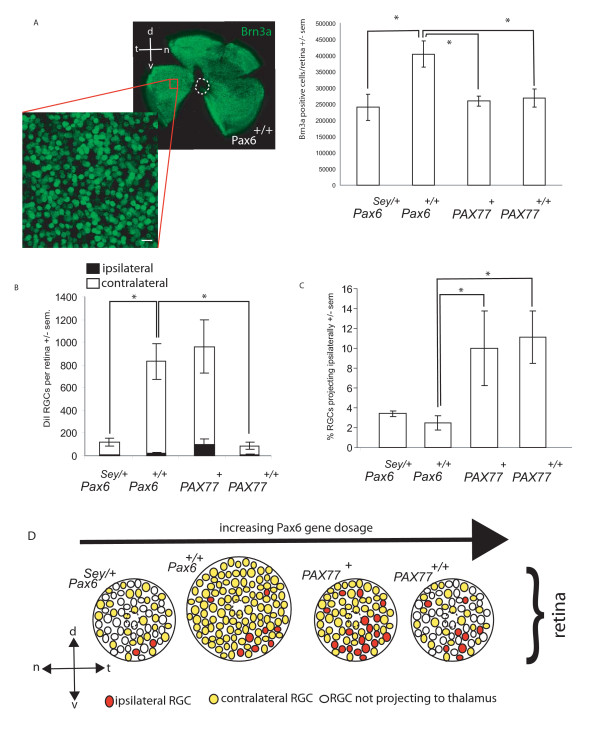
***Pax6/PAX6* gene dosage affects both the total number of RGCs, the number of RGC axons projected to the thalamus and the proportion that project ipsilaterally.** (A) Quantification of Brn3a-expressing cells in the retinae of E16.5 *Pax6*^*Sey*/+^, *Pax6*^+/+^, *PAX77*^+ ^and *PAX77*^+/+ ^embryos. Left, a wholemount retina immunostained with Brn3a antibody (green fluorescence). Dotted circle indicates location of optic nerve head. Boxed area shows location of one of the 160 μm × 160 μm sampling areas, in this case in ventro-temporal retina, used to calculate density of Brn3a-expressing cells (see Methods). The inset is an example of high power image showing individual Brn3a-expressing nuclei. Histogram on right shows the average total number of Brn3a-expressing cells in the retina Sample sizes: *Pax6*^*Sey*/+ ^n = 5; *Pax6*^+/+ ^n = 4; *PAX77*^+ ^n = 3; *PAX77*^+/+ ^n = 3. (B) Histogram showing the total numbers of RGCs per retina retrogradely labelled by unilateral injection of DiI into the dorsal thalamus of E16.5 embryos. Each bar is divided into ipsilateral (black) and contralateral (white) components. (C) Histogram showing the percentage of RGCs projecting ipsilaterally calculated from the data presented in (B). Sample sizes in B and C: *Pax6*^*Sey*/+ ^n = 4; *Pax6*^+/+ ^n = 9; *PAX77*^+ ^n = 5; *PAX77*^+/+ ^n = 4. In A-C, the embryo genotypes are indicated below each graph and are arranged in order of increasing *Pax6/PAX6 *gene dosage. In all histograms error bars are s.e.m.s with * indicating a significant difference from *Pax6*^+/+ ^embryos (p < 0.05 Mann-Whitney Rank Sum test). (E) Cartoon summarising the relationship between Pax6 gene dosage, and the number and location of RGCs generated by the retina (ovals), and whether they project ipsilaterally (filled red) contralaterally (filled yellow) or fail to project (unfilled) to the thalamus. Scale bar in A = 10 μm. Abbreviations: n, nasal; t, temporal, d, dorsal; v, ventral.

We studied whether the reduction in the number of RGCs labelled by DiI from the thalamus in *PAX77*^+/+ ^mutants could be accounted for by a reduction in the total number of RGCs in the mutant retina. In view of the interesting observation that there was a disproportionate reduction in the contralateral projection in *PAX77*^+/+ ^mutants, we extended the study to include embryos with a range of *Pax6 *gene dosage. We counted the total number of RGCs, marked by expression of Brn3a, in whole E16.5 retinae from *Pax6*^*Sey*/+^, *Pax6*^+/+^, *PAX77*^+^, and *PAX77*^+/+ ^embryos. This analysis showed that all three types of mutant retina produced about a third fewer Brn3a-expressing RGCs than the wild-type (Fig. 8A; this was significant p < 0.05; Mann-Whitney Rank Sum Test) although there was no significant difference between the mutant genotypes. This reduction in RGC number was accounted for by the reduction in retinal area in *Pax6*^*Sey*/+^, *PAX77*^+^, and *PAX77*^+/+ ^embryos as the density of Brn3a-expressing cells in the RGC layer and the depth of the RGC layer were similar in *Pax6*^*Sey*/+^, *Pax6*^+/+^, *PAX77*^+^, and *PAX77*^+/+ ^embryos (Table 2). There was no significant difference in the density of Brn3a-expressing cells between ventro-temporal and dorso-nasal retina for each genotype (Table 2), indicating that the relatively even distribution of RGCs over the retinal surface seen in wild-types is maintained in all three types of mutant.

**Table 2:** 

genotype	Retina area [μm^2 ^× 10^6^]	RGC layer depth [μm]	Density of Brn3a expressing cells in RGC layer [cells/μm^3 ^× 10^-3^]		Brn3a expressing cells/retina × 10^3^
				
			dn	vt	
*Pax6*^*Sey*/+^	1.74 ± 0.18	17.6 ± 1.4	7.9 ± 0.1	7.4 ± 0.6	241 ± 40
*Pax6*^+/+^	2.90 ± 0.24	16.4 ± 1.6	8.9 ± 1.1	8.4 ± 0.3	405 ± 41
*PAX77*^+^	1.87 ± 0.24	18.9 ± 3.0	7.5 ± 0.2	7.6 ± 0.2	260 ± 15
*PAX77*^+/+^	1.86 ± 0.20	17.9 ± 3.9	8.3 ± 0.3	8.5 ± 0.2	269 ± 28

Numbers and distributions of Brn3a expressing RGCs in *Pax6*^*Sey*/+^, *Pax6*^+/+^, *PAX77*^+^, and *PAX77*^+/+ ^embryos. Note that the reduced number of Brn3a-expressing cells in the *Pax6*^*Sey*/+^, *PAX77*^+^, and *PAX77*^+/+ ^embryos compared to *Pax6*^+/+ ^embryos (see Fig 7 A) appears to correspond to a reduction in retinal area [that is having smaller eyes] as opposed to changes in the density or distribution of Brn3a-expressing cells in the retina. All values mean ± s.e.m. Sample sizes: *Pax6*^*Sey*/+ ^n = 5; *Pax6*^+/+ ^n = 4; *PAX77*^+ ^n = 3; *PAX77*^+/+ ^n = 3. Abbreviations: dn, dorsonasal quadrant; vt, ventrotemporal quadrant.

**Figure 9:**
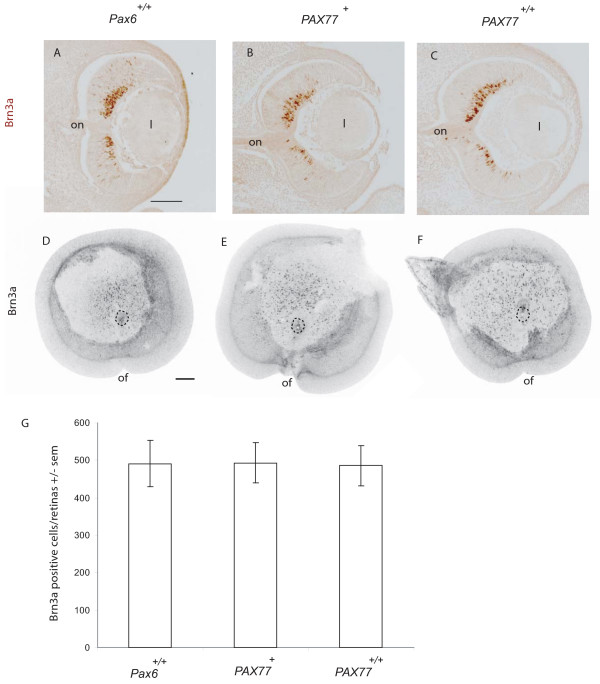
**The distribution and number of Brn3a expressing cells in the retina is not affected by Pax6 overexpression at E12.5.** (A-C) Brn3a (brown diaminobenzidine product) immunohistochemistry on coronal sections. (D-F) Brn3a immunofluorescence on wholemount retina with the lens removed viewed from the front and shown as a negative greyscale image for clarity. Dotted circle marks optic nerve head. (A,D) *Pax6*^+/+^, (B,E) *PAX77*^+^, (C,F) *PAX77*^+/+^. Note that in all genotypes the highest density of Brn3a expressing cells is located in the dorso-central retina. (G) Quantification of Brn3a positive cells showing that there is no significant difference in numbers in the three genotypes.*Pax6*^+/+ ^n = 3, *PAX77*^+ ^n = 3, *PAX77*^+/+ ^n = 4. Abbreviations: l, lens; on, optic nerve; of, optic fissure. Dorsal is at the top in all images. Scale bars: (A-C) and (D-F) 100 μm.

The reduced number of RGCs at E16.5 resulting from Pax6 overexpression raises the possibility that the onset of ganglion cell differentiation may be delayed. We therefore examined the number and distribution of Brn3a expressing cells at the onset of RGC differentiation [34,35] in E12.5 retinae of *Pax6*^+/+^, *PAX77*^+^, and *PAX77*^+/+ ^embryos (Fig 9). We found no differences in the distribution (Fig 9A–F) or number (Fig 9G) of Brn3a positive cells between the three genotypes. We conclude that the retinal axon guidance defects caused by Pax6 overexpression do not arise from delayed onset of RGC differentiation.

### The midline crossing behaviour of RGC axons at the optic chiasm is sensitive to Pax6 gene dosage

RGC axons are sorted into the optic tracts at the optic chiasm. In wild-type mice about 3% do not cross the midline and grow into the ipsilateral optic tract. The remaining 97% cross the midline and grow into the contralateral optic tract. In *PAX77*^+/+ ^embryos the increased levels of Pax6 correlate with an increase in the proportion of RGCs projecting ipsilaterally. In order to investigate the relationship between Pax6 and the ipsilateral projection in more detail we examined other lines of mice with different numbers of *Pax6*/*PAX6 *gene copies.

Small-eye heterozygote (*Pax6*^*Sey*/+^) embryos have only one functional copy of *Pax6 *[5]. Loss of one functional copy of *Pax6 *in *Pax6*^*Neu*/+ ^embryos produces a hypoplastic optic nerve [53]. Quantification of RGCs retrogradely labelled by unilateral DiI injection into the thalamus confirm that the total projection in *Pax6*^*Sey*/+ ^embryos is reduced to 12% (p < 0.05; Mann-Whitney Rank Sum Test) of that seen in wild-types (Fig 8B). In contrast to the *PAX77*^+/+ ^embryos, however, the reduction affects ipsilateral and contralateral projections equally and the proportion of RGCs projecting ipsilaterally is 3%, not significantly different to the 2.5% in the wild-type (Fig 8C; p > 0.05; Mann-Whitney Rank Sum Test). Reducing the number of functional *Pax6 *gene copies reduces the number of RGC axons projected by the retina to the thalamus but does not affect the proportion that cross the midline at the optic chiasm. Thus, reduction in the number of RGCs, which occurs in both *Pax6*^*Sey*/+ ^and *PAX77*^+/+ ^embryos, is not sufficient to explain the increased proportion of ipsilateral projections in *PAX77*^+/+ ^mutants, which must be regulated separately by gene dosage.

*PAX77*^+ ^hemizygotes [12] contain 5 to 7 copies of human *PAX6 *in addition to the two endogenous mouse *Pax6 *genes. The *Pax6*/*PAX6 *gene dosage in these embryos is therefore intermediate between wild-type embryos and *PAX77*^+/+ ^embryos [12]. Quantification of RGCs retrogradely labelled by unilateral DiI injection into the thalamus showed no significant difference in the overall numbers of RGCs projecting into the brain compared to wild-types (p > 0.05; Mann-Whitney Rank Sum Test; Fig 8B), even though the total number of Brn3a-expressing RGCs was less than in wild-types (Fig. 8A). The proportion of RGCs projecting ipsilaterally was about 10%, significantly more than the 2.5% seen in wild-types (p < 0.05; Mann-Whitney Rank Sum Test; Fig 8C). As described above, in *PAX77*^+/+ ^embryos there is a significant reduction of the total projection to 10% of its normal size (p < 0.05; Mann-Whitney Rank Sum Test; Figs 7F and 8B) of which 11% is ipsilateral, a significant increase compared to the wild type ipsilateral projection (p < 0.05; Mann-Whitney Rank Sum Test; Fig 8C). The tendency of ipsilaterally projecting RGCs to be most concentrated in the ventro-temporal retina, which has been well established in *Pax6*^+/+ ^embryos [eg [42,44,49]], was maintained in *Pax6*^*Sey*/+^, *PAX77*^+^, and *PAX77*^+/+ ^embryos (Table 3) irrespective of the changes in the total number or proportion of ipsilaterally projecting RGCs. These results, summarised in Fig 8D, show that increasing the number of functional *PAX6 *gene copies increases the proportion of RGC axons projecting ipsilaterally independently of its effect on the total numbers of RGCs whose axons reach the thalamus.

**Table 3:** 

genotype	Distribution of RGCs projecting ipsilaterally from each retinal quadrant (expressed as % of total projection to thalamus)				% of ipsilateral projection originating from vt retina
		
	dn	dt	vn	**vt**	
*Pax6*^*Sey*/+^	0.53 ± 0.26	0.23 ± 0.01	0.40 ± 0.20	**1.44 ± 0.72**	44 ± 25
*Pax6*^+/+^	0.22 ± 0.06	0.39 ± 0.12	0.33 ± 0.17	**1.16 ± 0.47**	45 ± 10
*PAX77*^+^	1.03 ± 0.46	1.59 ± 0.42	1.27 ± 0.43	**4.01 ± 2.32**	34 ± 11
*PAX77*^+/+^	1.24 ± 0.71	2.30 ± 1.57	0	**5.84 ± 2.70**	44 ± 18

Distributions of RGCs projecting ipsialterally to the thalamus in *Pax6*^*Sey*/+^, *Pax6*^+/+^, *PAX77*^+^, and *PAX77*^+/+ ^embryos in the four retinal quadrants. Note that in all genotypes the ipsilaterally projecting RGCs are enriched in the ventrotemporal (vt) quadrant (shown in bold) compared to the dorsonasal (dn), ventronasal (vn), and dorsotemporal (dt) quadrants regardless of the overall size of the ipsilateral projection (shown in Fig 8 B,C). All values mean ± s.e.m. Sample sizes: *Pax6*^*Sey*/+ ^n = 4; *Pax6*^+/+ ^n = 9; *PAX77*^+ ^n = 5; *PAX77*^+/+ ^n = 4.

## Discussion

We analyzed the effects of Pax6 overexpression on the development of the neural retina and on its ability to project axons to the brain. Our results indicate that, in the eye of *PAX77*^+/+ ^embryos, (1) development proceeds normally until about E12.5, (2) the first defects, which are of the optic nerve and the growth and navigation of RGC axons, emerge at E14.5–E16.5, (3) major retinal cell types are generated and (4) there is progressive retinal dysplasia and microphthalmia from E18.5 into postnatal life. The globe and retina fail to grow and by P4 the vitreous has almost disappeared leaving the retina in contact with the lens. Retinal lamination is also affected with the appearance of many rosettes and abnormal horizontal cells. One clear conclusion from these findings is that while both over- and under-expression of Pax6 cause microphthalmia, the underlying mechanisms are different. Microphthalmia in *Pax6*^*Sey*/+ ^mice is thought to be caused by primary defects of the lens and anterior structures occurring at the time of initial eye formation rather than with later retinal defects [8,9].

### Possible causes of intraretinal RGC axonal defects

During normal development RGC axons initially grow straight towards the optic nerve head. Overexpression of Pax6 does not prevent RGC axons converging on the optic nerve head altogether, but does cause a substantial proportion to take a less direct route than normal. The intraretinal navigation defects occur before E16.5, when the morphology of the *PAX77*^+/+ ^retina is not grossly abnormal, making it less likely that the distorted RGC axons are a secondary consequence of retinal dysplasia. Intraretinal axon navigation defects have been reported in mice lacking the secreted axon guidance molecules Slit1 and/or Slit2 [54] or the cell surface receptors EphB2 and EphB3 [55], or netrin1 and its receptor DCC (deleted in colorectal cancer) [56]. Although these loss-of-function phenotypes do not precisely phenocopy the *PAX77*^+/+ ^phenotype reported here, it is possible that Pax6 normally regulates the expression of one or more of these molecules and that its overexpression alters their normal balance resulting in the intraretinal axon guidance defects we observed. As Pax6 is expressed by RGCs themselves, and by other retinal cell types, it is possible that the intraretinal pathfinding errors reflect sensitivity of the RGC growth cone and/or the environment through which it navigates to levels of Pax6. It has recently been postulated that the lens directs RGC axons towards the optic nerve head by secreting repulsive Slit molecules [54]. As Pax6 plays a role in the formation of the lens [10], and overexpressing Pax6 causes defects in lens development [57-59], it is quite possible that overexpression of Pax6 in the lens is directly responsible for a component of the intraretinal misrouting. The proper formation of the optic nerve head is also important for intraretinal axon navigation [56] and its altered structure in *PAX77*^+/+ ^embryos could influence axon navigation.

### Possible causes of RGC axonal defects at the optic chiasm

Retinal axon navigation at the optic chiasm is controlled by genes ranging from transcription factors [60] to cell surface and secreted proteins [61]. Manipulating a small subset of these genes produces specific alterations to chiasm organisation (for example see [53,62-66]). This complex genetic control may underlie variations in the details of chiasm construction between vertebrate species. In mice the vast majority of retinal axons cross the ventral midline at the optic chiasm and join the contralateral optic tract growing towards their targets in the thalamus and superior colliculus whereas a minority do not cross and join the ipsilateral tract [67]. To date only a small number of transcription factors have been identified which control the balance between ipsilateral and contralateral projections (*i.e*. [62,66,68,69]). A study of chick retina identified a gradient of Pax6 expression levels consistent with a role in topographic mapping of RGC axons via regulation of the expression of the axon guidance receptor EphB2 [70]. As signalling between EphB receptors and their ephrinB ligands have been implicated in axon crossing behaviour at the chiasm [64], it is possible that the regulation of EphB by Pax6 is conserved between chick and mouse and that overexpressing Pax6 alters the expression of EphB receptors on RGC growth cones and influences their midline crossing behaviour at the chiasm.

To investigate the relationship between Pax6 levels and RGC axon navigation at the optic chiasm we examined an allelic series of genotypes with different *Pax6/PAX6 *gene dosage. Overexpression of Pax6 resulted in an increase in the proportion of RGCs projecting ipsilaterally combined with either no change in the total number projecting (as in the *PAX77*^+ ^phenotype) or with a reduction in the total numbers projecting (as in the *PAX77*^+/+ ^phenotype). Conversely a reduction in the total numbers projecting occurred without changing the proportion of the ipsilateral projection in the *Pax6*^*Sey*/+ ^phenotype. This provides strong evidence that the increased proportion of ipsilaterally projecting axons in *PAX77*^+/+ ^embryos is not simply a consequence of a reduced total projection. As Pax6 is expressed by RGCs at the time their axons navigate the midline but not by chiasm cells at the midline it is likely that Pax6 levels in the RGCs themselves are important in defining the navigational properties of their growth cones. Pax6 overexpression might increase RGC axon growth cones' sensitivity to repulsive guidance cues at the chiasm [64], therefore reducing the proportion that will cross the midline and project contralaterally. This might occur either directly by regulation of axon guidance molecules, such as EphB1 [64,70], or indirectly by regulation of transcription factors, such as Zic2 [62], which program an RGC to project ipsilaterally. In either case this is the first evidence that Pax6 acts as an 'ipsilateral determinant' in its own right.

Brn3a is expressed by a large population of RGCs innervating the principal retinothalamic and retinocollicular pathway [71] so is a useful marker for determining whether the size of the population of RGCs that would normally project to the thalamus is affected when *Pax6/PAX6 *dosage is altered. RGCs projecting to ipsilateral targets in the thalamus and superior colliculus do not express Brn3a whereas contralateral projecting RGCs do, indicating that Brn3a expressing RGCs tend to project contralaterally [71]. Embryos either underexpressing or overexpressing *Pax6/PAX6 *have similar numbers of Brn3a expressing RGCs but different numbers projecting to the ipsilateral and contalateral thalamus indicating that in our models alterations of the size of the ipsilateral or contralateral projection cannot be explained by variations in the numbers of cells expressing Brn3a. The total numbers of Brn3a-expressing RGCs produced by *Pax6*^*Sey*/+^, *PAX77*^+^, and *PAX77*^+/+ ^embryos at E16.5 were very similar to one another, although in all cases about 30% less than in *Pax6*^+/+ ^embryos. The earliest RGC projection is a transient ipsilateral projection from the dorsocentral retina at E12.5 [72]. As Pax6 overexpression does not affect the number or dorsocentral location of Brn3a expressing RGCs at E12.5, it is extremely unlikely that the increased ipsilateral projection following Pax6 overexpression is simply a consequence of delayed retinal development. The 90% reduction in the total numbers of RGCs projecting to the thalamus in *Pax6*^*Sey*/+ ^and *PAX77*^+/+ ^embryos cannot be accounted for by a failure to generate RGCs but must be caused by axons failing to get to the thalamus, for example by wandering around in the retina, as seen in *PAX77*^+/+ ^embryos. It is possible that some RGCs fail to project axons out of the retina because they have a defect in axonogenesis like that seen when RGCs lack the transcription factor Brn3b [73].

### Possible causes of early defects of optic nerve head

Pax6 is initially co-expressed with Pax2 in the early optic stalk before becoming restricted to the optic cup by E12.5. Baumer et al. [74] have shown that maintaining the expression of Pax6 in the optic stalk after E12.5, by expressing Pax6 under the control of a Pax2-upstream promoter fragment, results in the appearance of retinal pigmented epithelium (RPE) in the optic disc, which is one of the earliest abnormalities detected in *PAX77*^+/+ ^E14.5 embryos. Although we did not detect Pax6 in the optic stalk of *PAX77*^+/+ ^mice after E12.5, it is possible that its overexpression causes a slight delay in its downregulation in the optic stalk. Such a delay could be responsible for the ectopic presence of RPE and retinal cells in the optic disc.

The malformation of the optic disc in *PAX77*^+/+ ^embryos could arise as a consequence of the abnormal development of RGC axons. Alternatively, the defects of RGC axon guidance might be secondary to primary defects at the optic nerve head. In this study we show that, at E16.5, RGCs display axon guidance defects and the levels of *Shh *mRNA, specifically expressed by the RGCs [40,41], are slightly lower than normal. Previous studies have shown that Shh, expressed by the RGCs, is implicated in retinal lamination and RGC axon guidance. Conditional inactivation of *Shh *in RGCs causes a complete loss of optic disc astrocyte precursor cells, resulting in defective axon guidance in the retina, as well as conversion of the neuroepithelial cells in the optic stalk to pigmented cells [40]. Loss of RGC-derived Shh also causes extensive disorganization of the retina with the appearance of many rosettes in the outer nuclear layer [41]. Thus, the slight decrease in Shh expression found in the mutant retina at E16.5 could reflect abnormalities in RGC differentiation and could contribute to the defects in RGC axon guidance and retinal lamination observed in the PAX77 retina.

In addition, another study has shown that specific ablation of newly formed RGCs results in a decrease of retinal progenitor cell proliferation and abnormally thin retinal layers in the postnatal eye [75]. Thus, a defect in RGC differentiation could also account for the reduced size of the retina in *PAX77*^+/+^mice. The fact that major retinal defects appear after RGC defects in axon guidance and Shh expression supports the hypothesis that microphthalmia and retinal dysplasia in *PAX77*^+/+ ^eyes could be secondary to earlier abnormalities in RGC differentiation caused by Pax6 overexpression.

## Conclusion

Higher than normal levels of Pax6 produce complex phenotypes in which several features of eye morphogenesis are affected. Both increased (present study and [12]) and decreased [76-80]*Pax6 *gene dosage result in defective eye development, but the effects are quite different. Whereas defects of *Pax6*^+/- ^mice occur mainly in the anterior segment of the eye [76-80], we show here that overexpression of Pax6 causes major defects of retinal development. Increased Pax6 levels also affect the projection of retinal axons. Since retinal axon navigation generally follows eye morphogenesis it is possible that a component of the intraretinal guidance errors we detect is a secondary consequence of alterations in retinal, lens, and/or optic disc anatomy or molecular composition. The navigation errors we detect at the chiasm are likely to reflect a requirement for normal Pax6 levels in programming the responses of RGCs to guidance cues at the chiasm.

## Methods

### Mice

PAX77 hemizygous mice [12], designated *PAX77*^+^, carry 5 to 7 copies of a 420 Kb human *PAX6 *YAC (Y593) with all copies integrated at the same locus. We refer to the array of integrated YAC Y593 copies as the *PAX77 *transgene. PAX77 homozygous mice, designated *PAX77*^+/+^, carry 10 to 14 copies of human *PAX6 *and were genotyped by fluorescent *in situ *hybridization using the Fat5 probe as described in Schedl et al. (1996) [12]. As no abnormalities are observed in animals carrying the transgene on a *Pax6*^*Sey*/+ ^heterozygote background Schedl et al[12] concluded that the ocular abnormalities in PAX77 mice are due to overexpression of PAX6 and not to overexpression of a second gene encoded by the YAC. All mice were maintained on an albino CD1 background. The morning of the vaginal plug was deemed E0.5. The first 24 h after birth was deemed P0. Animal care followed institutional guidelines and UK Home Office regulations.

### Histology

For each stage and each genotype, at least 2 samples were fixed in 4% paraformaldehyde : 2% glutaraldehyde, dehydrated to 100% ethanol and embedded in cold-polymerizing resin (Technovit 7100, Kulzer Histo-Technik). Sections were cut (5 μm) and stained with cresyl violet. Sections of whole embryos were cut horizontally and sections of postnatal eyes were cut parallel to the optic nerve.

### BrdU/IdU analysis of cell-cycle parameters

The BrdU/IdU double labeling protocol is detailed in Martynoga et al.(2005) [15]. In brief, at T = 0 h the pregnant mouse receives an injection of IdU which labels cells going through S-phase from the beginning of the experiment. At T = 1.5 h the female receives an injection of BrdU and the embryos are fixed shortly after (T = 2 h). Thus, the BrdU labels cells which are in S-phase at the end of the experiment; this is the S fraction (S_cells_). The cells labeled only by the IdU but not by the BrdU are cells which have left S phase in the interval of 1.5 h between the two injections. This is the leaving fraction (L_cells_). The cell cycle length (Tc) and the length of S-phase (Ts) can be calculated using the equations of Shibui et al.(1989) [14]: Ts = Ti/(L_cells_/S_cells_) and Tc = (S_cells_/P_cells_), where Ti is the interval between the two injections and P_cells _is the total number of proliferating cells. We made the assumption that all progenitor cells of the retina are proliferating. The growth fraction was not determined in this experiment.

Pregnant females were injected intra-peritoneally with 200 ml of 10 mg/ml (in 0.9% NaCl) IdU (Sigma), then 1.5 h later with the same dose of BrdU (Sigma) and they were sacrificed after 30 min. 10 μm wax sections were immunostained with mouse anti-BrdU/IdU (Becton Dickinson, clone B44, 1:100) and rat anti-BrdU (Abcam, clone BU1/75, 1:100). Cells were counted in 100 μm wide sampling boxes in the distal and proximal retina of 3 wild-type and 3 *Pax77*^+/+ ^E12.5 embryos. Each cell count was repeated on at least 3 non-adjacent sections from each embryo.

### Immunohistochemistry

Whole embryos or postnatal eyes were fixed in 4% paraformaldehyde and either processed to wax or cryoprotected in 15% and 30% sucrose and frozen.

Immunostaining for Pax6, glial fibrillary acidic protein (GFAP), Brn3a, Islet1, bromodeoxyuridine (BrdU), Syntaxin and N-Cadherin (Ncad) was performed on 10 μm paraffin sections as described in Martynoga et al. (2005) [15]. For studies with BrdU, pregnant females were injected intra-peritoneally with 200 μl of 10 mg/ml BrdU (in 0.9% NaCl; Sigma) and were sacrificed after 1 hour. Immunostaining for Chx10 and Pax2 was performed on 10 μm frozen sections as described in Martynoga et al. (2005) [15]. R-Cadherin (Rcad) immunostaining was performed on frozen sections as described in [81]. Sections of whole embryos were cut horizontally and sections of postnatal eyes were cut parallel to the optic nerve.

For whole-mount immunofluorescence, dissected retinae from E16.5 embryos were incubated in blocking buffer (20% goat serum, 0.2% Triton-X100 in phosphate buffered saline [PBS]) for 20 min, followed by anti-neurofilament or anti-L1 antibody overnight at 4°C. For neurofilament detection, sections were rinsed in 0.2% Triton-X100 in PBS, and incubated with an Alexa488-conjugated anti-rabbit secondary (Molecular Probes, Inc). For L1 detection, a biotinylated secondary antibody was used with Alexa488-conjugated streptavidin (Molecular Probes, Inc).

Primary antibodies were: Pax6 (1:400, DSHB), GFAP (1:50, Dako), Brn3a (1:300, Chemicon; MAB1585), Islet1 (1:50, DSHB), Ncad (1:500, BD), BrdU (1:200, Becton Dickinson), Syntaxin (1:100, Santa-Cruz sc-12736), Chx10 (1:1000, a gift from C. Cepko), Pax2 (1:200, Cambridge Bioscience), Rcad (1:100, a gift from M. Takeichi), Neurofilament (1:100, BIOMOL, USA; NA1297), L1 (1:50, Chemicon; MAB5272).

### Quantification of Brn3a-expressing cells in the retina

Retinae were removed from heads previously fixed in 4% paraformaldehyde and an orientating cut made in the ventral retina of E16.5 embryos. Retinae were boiled in 10 mM sodium citrate buffer pH6.0 for 10 minutes and blocked in 5% goat serum, 0.2% BSA, 0.1% Triton-X1000 in PBS at room temperature before incubating overnight at 4°C in primary antibody [Brn3a Chemicon; MAB1585)] diluted 1:300 in blocking buffer. After washing extensively in 0.1% Triton-X1000 in PBS at room temperature retinae were incubated overnight at 4°C with an Alexa488-conjugated anti-mouse secondary (Molecular Probes, Inc), washed extensively, and mounted with the RGC layer uppermost in 90% glycerol containing MOWIOL and DABCO. For E16.5 retinae, a Leica TCS NT confocal microscope was used to acquire (1) a low power image of the entire retina for calculation of retinal area [see Fig 8A], (2) to scan through the entire Brn3a-expressing RGC layer at high power and measure its depth and (3) to collect a series of optical sections for sampling within the RGC layer.

Sections were 160 μm × 160 μm and were collected at 0.16 μm intervals through 8 μm of retinal depth. The boxed area in Fig 8A shows an example of a sampling area in ventrotemporal retina. We used a stereological approach to calculate the density of RGCs within the sampling volume: optical sections comprising the upper and lower 4 μm of the total 8 μm stack were combined separately to generate images like the high power inset shown in Fig 8A. Simply counting the number of Brn3a-expressing nuclei in each image would give an overestimate of nuclear density in each 160 μm × 160 μm × 4 μm volume as nuclei protruding into the sampling volume both from above and from below would be included in the count. To avoid overestimating the nuclear density, we only counted nuclei protruding into the sampling volume from one side while excluding those that protruded from the other. This was done by using Adobe Photoshop to pseudo-colour the nuclei in the upper stack red and those in the lower stack green and then merging the two images. Nuclei appearing yellow [red and green combined] were present in both upper and lower stacks and so were excluded from the count. This gave an accurate number of Brn3a-expressing nuclei present in the sampling volume and was used to calculate their density. For each retina the density was calculated in the dorso-nasal and ventro-temporal retina. The number of Brn3a-expressing cells in the retina was calculated by multiplying the retinal area × depth of RGC layer × density of Brn3a-expressing cells in RGC layer. For E12.5 retinae, where the density of RGCs is much lower, a Leica TCS NT confocal microscope was used to acquire a scan through the whole retina and total numbers of Brn3a positive cells were counted.

### Tract-Tracing

Tract-tracing was performed as described in Pratt et al. (2006) [65]. Embryonic heads were fixed at 4°C in 4% paraformaldehyde in PBS overnight. DiI crystals (Molecular probes, USA) were either (1) placed in the optic cup of one eye after removal of the lens or (2) placed in a line over the dorsal thalamus on one side to label axons navigating the optic tract. Heads were returned to 4% paraformaldehyde in PBS in the dark at room temperature for about six weeks to allow tracers to diffuse along axons. In some cases the retinae were removed from the head, cleared in 9:1 glycerol:PBS, and imaged as wholemounts using a Zeiss Axiovert confocal LSM 510 microscope (Zeiss, Germany). In other cases heads were sectioned (200 μm) with a vibratome, cleared in 9:1 glycerol:PBS containing the nuclear counterstain TOPRO3 (1.0 μM, Molecular Probes, USA), mounted in Vectashield (Vector Laboratories, USA), and imaged using an epifluorescence microscope and digital camera (Leica Microsystems, Germany) or a TCS NT confocal microscope (Leica Microsystems, Germany). For quantification of RGC projections the total number of DiI labelled RGCs in each retina was counted in serial vibratome sections using an epifluorescence microscope as follows. Each section was viewed under epifluorescence using a x20 objective at which magnification DiI labelled RGC cell bodies are easily resolved. An eyepiece graticule was used to divide the retina into 50 μm wide bins. Within each bin DiI labelled RGC bodies were counted by focussing through the thickness of the section and counting labelled cell bodies as they came into focus. Individual bin counts were then summed to give totals for RGCs projecting ipsilaterally and contralaterally to the thalamic DiI injection site in each embryo and these totals were used to generate data presented in Fig 7, Fig 8, and Table 3. The numbers of E16.5 embryos used for quantification were as follows: *Pax6*^*Sey*/+ ^n = 4; *Pax6*^+/+ ^n = 9; *PAX77*^+ ^n = 5; *PAX77*^+/+ ^n = 4. DiI appears orange and TOPRO3 appears red in epifluorescence images and in confocal images DiI appears red and TOPRO3 appears blue.

### Quantitative reverse transcription-PCR (qRT-PCR)

For each sample RNA was extracted from a pool of retinae from a wild-type or *PAX77*^+/+ ^litter using Qiagen RNeasy kit (Qiagen, USA). cDNA synthesis was performed as described in [82]. qRT-PCR was performed on cDNA from E14.5 and E16.5 retinae with the following primer pairs (n = 3 litters): *Ncam (*5'-GACCATCAGGAATGTGGA-3' and 5'-AGGCTTCACAGGTCAGAGT-3'; 179 bp product); *R-cadherin *(5'-CAGTGAAACAGGGGACATC-3' and 5'-ATACGGTTCTCAGGAACCTC-3'; 216 bp product); *Ngn2 *(5'-CAAACTTTCCCTCTCTGATG-3' and 5'-CATTCAACCCTTACAAAAGC-3' ; 197 bp product);*L1 *(5'-ACCCTGAGGCATTACACCTG-3' and 5'-CAACTGCTCTTTGCTTTCCC-3'; 140 bp product); *Six3 *(5'-CTGGAGAACCACAAGTTCAC-3' and 5'-GATCCTGCAGGTACCACTC-3'; 232 bp product); mouse *Pax6 *(5'-AACACCAACTCCATCAGTTC-3' and 5'-ATCTGGATAATGGGTCCTCT-3'; 153 bp product); human + mouse *Pax6 *(5'-TAGCGAAAAGCAACAGATG-3' and 5'-TCTATTTCTTTGCAGCTTCC-3'; 250 bp product); *Shh *(5'-CCCTTTAGCCTACAAGCAGT-3' and 5'-CCACTGGTTCATCACAGAG-3'; 232 bp product) and *GAPDH *(5'-GGGTGTGAACCACGAGAAAT-3' and 5'-CCTTCCACAATGCCAAAGTT-3'; 121 bp product). Quantitative RT-PCR was performed using Qiagen Quantitect SYBR Green PCR Kit (Qiagen, USA) and a DNA Engine Opticon Continuous Fluorescence Detector (GRI, UK). The abundance of each transcript in the original RNA sample was extrapolated from PCR reaction kinetics using Opticon software and normalised to the level of GAPDH transcript.

## Authors' contributions

MM and TP designed and supervised the study, carried out some of the experiments and wrote the paper, ML carried out some of the experiments, GJ participated in the analysis and in writing the paper, DJP participated in design and supervision and in writing the paper. All authors read and approved the final manuscript.

## References

[B1] Walther C, Gruss P (1991). Pax-6, a murine paired box gene, is expressed in the developing CNS. Development.

[B2] Grindley JC, Davidson DR, Hill RE (1995). The role of Pax-6 in eye and nasal development. Development.

[B3] Marquardt T, Ashery-Padan R, Andrejewski N, Scardigli R, Guillemot F, Gruss P (2001). Pax6 is required for the multipotent state of retinal progenitor cells. Cell.

[B4] Baumer N, Marquardt T, Stoykova A, Ashery-Padan R, Chowdhury K, Gruss P (2002). Pax6 is required for establishing naso-temporal and dorsal characteristics of the optic vesicle. Development.

[B5] Hill RE, Favor J, Hogan BL, Ton CC, Saunders GF, Hanson IM, Prosser J, Jordan T, Hastie ND, van Heyningen V (1991). Mouse small eye results from mutations in a paired-like homeobox-containing gene. Nature.

[B6] Collinson JM, Hill RE, West JD (2000). Different roles for Pax6 in the optic vesicle and facial epithelium mediate early morphogenesis of the murine eye. Development.

[B7] Quinn JC, West JD, Hill RE (1996). Multiple functions for Pax6 in mouse eye and nasal development. Genes Dev.

[B8] van Raamsdonk CD, Tilghman SM (2000). Dosage requirement and allelic expression of PAX6 during lens placode formation. Development.

[B9] Collinson JM, Quinn JC, Buchanan MA, Kaufman MH, Wedden SE, West JD, Hill RE (2001). Primary defects in the lens underlie complex anterior segment abnormalities of the Pax6 heterozygous eye. Proc Natl Acad Sci U S A.

[B10] Ashery-Padan R, Marquardt T, Zhou X, Gruss P (2000). Pax6 activity in the lens primordium is required for lens formation and for correct placement of a single retina in the eye. Genes Dev.

[B11] Simpson TI, Price DJ (2002). Pax6; a pleiotropic player in development. Bioessays.

[B12] Schedl A, Ross A, Lee M, Engelkamp D, Rashbass P, van Heyningen V, Hastie ND (1996). Influence of PAX6 gene dosage on development: overexpression causes severe eye abnormalities. Cell.

[B13] Manuel M, Georgala PA, Carr CB, Chanas S, Kleinjan DA, Martynoga B, Mason JO, Molinek M, Pinson J, Pratt T, Quinn JC, Simpson TI, Tyas DA, van Heyningen V, West JD, Price DJ (2007). Controlled overexpression of Pax6 in vivo negatively autoregulates the Pax6 locus, causing cell-autonomous defects of late cortical progenitor proliferation with little effect on cortical arealization. Development.

[B14] Shibui S, Hoshino T, Vanderlaan M, Gray JW (1989). Double labeling with iodo- and bromodeoxyuridine for cell kinetics studies. J Histochem Cytochem.

[B15] Martynoga B, Morrison H, Price DJ, Mason JO (2005). Foxg1 is required for specification of ventral telencephalon and region-specific regulation of dorsal telencephalic precursor proliferation and apoptosis. Dev Biol.

[B16] Barber AJ, Nakamura M, Wolpert EB, Reiter CE, Seigel GM, Antonetti DA, Gardner TW (2001). Insulin rescues retinal neurons from apoptosis by a phosphatidylinositol 3-kinase/Akt-mediated mechanism that reduces the activation of caspase-3. J Biol Chem.

[B17] Bonfanti L, Strettoi E, Chierzi S, Cenni MC, Liu XH, Martinou JC, Maffei L, Rabacchi SA (1996). Protection of retinal ganglion cells from natural and axotomy-induced cell death in neonatal transgenic mice overexpressing bcl-2. J Neurosci.

[B18] Chong NH, Alexander RA, Barnett KC, Bird AC, Luthert PJ (1999). An immunohistochemical study of an autosomal dominant feline rod/cone dysplasia (Rdy cats). Exp Eye Res.

[B19] Cusato K, Bosco A, Rozental R, Guimaraes CA, Reese BE, Linden R, Spray DC (2003). Gap junctions mediate bystander cell death in developing retina. J Neurosci.

[B20] Daly FJ, Sandell JH (2000). Inherited retinal degeneration and apoptosis in mutant zebrafish. Anat Rec.

[B21] Diaz B, Pimentel B, de Pablo F, de La Rosa EJ (1999). Apoptotic cell death of proliferating neuroepithelial cells in the embryonic retina is prevented by insulin. Eur J Neurosci.

[B22] Gong H, Amemiya T, Takaya K (2001). Retinal changes in magnesium-deficient rats. Exp Eye Res.

[B23] Kurimoto T, Miyoshi T, Suzuki A, Yakura T, Watanabe M, Mimura O, Fukuda Y (2003). Apoptotic death of beta cells after optic nerve transection in adult cats. J Neurosci.

[B24] Li Y, Schlamp CL, Nickells RW (1999). Experimental induction of retinal ganglion cell death in adult mice. Invest Ophthalmol Vis Sci.

[B25] Nagai-Kusuhara A, Nakamura M, Mukuno H, Kanamori A, Negi A, Seigel GM (2007). cAMP-responsive element binding protein mediates a cGMP/protein kinase G-dependent anti-apoptotic signal induced by nitric oxide in retinal neuro-glial progenitor cells. Exp Eye Res.

[B26] Pisani F, Pedale S, Macaione V, Torre V, Oteri G, Avanzini G, Ientile R (2001). Neuroprotective effects of lamotrigine and remacemide on excitotoxicity induced by glutamate agonists in isolated chick retina. Exp Neurol.

[B27] Poon HK, Tso MO, Lam TT (2000). c-Fos protein in photoreceptor cell death after photic injury in rats. Invest Ophthalmol Vis Sci.

[B28] Quigley HA, Nickells RW, Kerrigan LA, Pease ME, Thibault DJ, Zack DJ (1995). Retinal ganglion cell death in experimental glaucoma and after axotomy occurs by apoptosis. Invest Ophthalmol Vis Sci.

[B29] Sale A, Cenni MC, Ciucci F, Putignano E, Chierzi S, Maffei L (2007). Maternal Enrichment during Pregnancy Accelerates Retinal Development of the Fetus. PLoS ONE.

[B30] Zeevalk GD, Nicklas WJ (2000). Lactate prevents the alterations in tissue amino acids, decline in ATP, and cell damage due to aglycemia in retina. J Neurochem.

[B31] Lotto RB, Asavaritikrai P, Vali L, Price DJ (2001). Target-derived neurotrophic factors regulate the death of developing forebrain neurons after a change in their trophic requirements. J Neurosci.

[B32] Smith RS, Smith RS, John SWM, Nishina PM and Sundberg JP (2002). Choroid, Lens and Vitreous. Mouse Eye Anatomy, Pathology and Biomethods.

[B33] Otteson DC, Shelden E, Jones JM, Kameoka J, Hitchcock PF (1998). Pax2 expression and retinal morphogenesis in the normal and Krd mouse. Dev Biol.

[B34] Xiang M, Zhou L, Macke JP, Yoshioka T, Hendry SH, Eddy RL, Shows TB, Nathans J (1995). The Brn-3 family of POU-domain factors: primary structure, binding specificity, and expression in subsets of retinal ganglion cells and somatosensory neurons. J Neurosci.

[B35] Pan L, Yang Z, Feng L, Gan L (2005). Functional equivalence of Brn3 POU-domain transcription factors in mouse retinal neurogenesis. Development.

[B36] Philips GT, Stair CN, Young Lee H, Wroblewski E, Berberoglu MA, Brown NL, Mastick GS (2005). Precocious retinal neurons: Pax6 controls timing of differentiation and determination of cell type. Dev Biol.

[B37] Rowan S, Cepko CL (2004). Genetic analysis of the homeodomain transcription factor Chx10 in the retina using a novel multifunctional BAC transgenic mouse reporter. Dev Biol.

[B38] Honjo M, Tanihara H, Suzuki S, Tanaka T, Honda Y, Takeichi M (2000). Differential expression of cadherin adhesion receptors in neural retina of the postnatal mouse. Invest Ophthalmol Vis Sci.

[B39] Matsunaga M, Hatta K, Takeichi M (1988). Role of N-cadherin cell adhesion molecules in the histogenesis of neural retina. Neuron.

[B40] Dakubo GD, Wang YP, Mazerolle C, Campsall K, McMahon AP, Wallace VA (2003). Retinal ganglion cell-derived sonic hedgehog signaling is required for optic disc and stalk neuroepithelial cell development. Development.

[B41] Wang YP, Dakubo G, Howley P, Campsall KD, Mazarolle CJ, Shiga SA, Lewis PM, McMahon AP, Wallace VA (2002). Development of normal retinal organization depends on Sonic hedgehog signaling from ganglion cells. Nat Neurosci.

[B42] Andrews GL, Mastick GS (2003). R-cadherin is a Pax6-regulated, growth-promoting cue for pioneer axons. J Neurosci.

[B43] Demyanenko GP, Maness PF (2003). The L1 cell adhesion molecule is essential for topographic mapping of retinal axons. J Neurosci.

[B44] Edelman GM, Jones FS (1995). Developmental control of N-CAM expression by Hox and Pax gene products. Philos Trans R Soc Lond B Biol Sci.

[B45] Walsh FS, Doherty P (1997). Neural cell adhesion molecules of the immunoglobulin superfamily: role in axon growth and guidance. Annu Rev Cell Dev Biol.

[B46] Zhu CC, Dyer MA, Uchikawa M, Kondoh H, Lagutin OV, Oliver G (2002). Six3-mediated auto repression and eye development requires its interaction with members of the Groucho-related family of co-repressors. Development.

[B47] Lagutin O, Zhu CC, Furuta Y, Rowitch DH, McMahon AP, Oliver G (2001). Six3 promotes the formation of ectopic optic vesicle-like structures in mouse embryos. Dev Dyn.

[B48] Dyer MA (2003). Regulation of proliferation, cell fate specification and differentiation by the homeodomain proteins Prox1, Six3, and Chx10 in the developing retina. Cell Cycle.

[B49] Goudreau G, Petrou P, Reneker LW, Graw J, Loster J, Gruss P (2002). Mutually regulated expression of Pax6 and Six3 and its implications for the Pax6 haploinsufficient lens phenotype. Proc Natl Acad Sci U S A.

[B50] Matter-Sadzinski L, Puzianowska-Kuznicka M, Hernandez J, Ballivet M, Matter JM (2005). A bHLH transcriptional network regulating the specification of retinal ganglion cells. Development.

[B51] Scardigli R, Baumer N, Gruss P, Guillemot F, Le Roux I (2003). Direct and concentration-dependent regulation of the proneural gene Neurogenin2 by Pax6. Development.

[B52] Scardigli R, Schuurmans C, Gradwohl G, Guillemot F (2001). Crossregulation between Neurogenin2 and pathways specifying neuronal identity in the spinal cord. Neuron.

[B53] Deiner MS, Sretavan DW (1999). Altered midline axon pathways and ectopic neurons in the developing hypothalamus of netrin-1- and DCC-deficient mice. J Neurosci.

[B54] Thompson H, Camand O, Barker D, Erskine L (2006). Slit proteins regulate distinct aspects of retinal ganglion cell axon guidance within dorsal and ventral retina. J Neurosci.

[B55] Birgbauer E, Cowan CA, Sretavan DW, Henkemeyer M (2000). Kinase independent function of EphB receptors in retinal axon pathfinding to the optic disc from dorsal but not ventral retina. Development.

[B56] Deiner MS, Kennedy TE, Fazeli A, Serafini T, Tessier-Lavigne M, Sretavan DW (1997). Netrin-1 and DCC mediate axon guidance locally at the optic disc: loss of function leads to optic nerve hypoplasia. Neuron.

[B57] Duncan MK, Cvekl A, Li X, Piatigorsky J (2000). Truncated forms of Pax-6 disrupt lens morphology in transgenic mice. Invest Ophthalmol Vis Sci.

[B58] Duncan MK, Kozmik Z, Cveklova K, Piatigorsky J, Cvekl A (2000). Overexpression of PAX6(5a) in lens fiber cells results in cataract and upregulation of (alpha)5(beta)1 integrin expression. J Cell Sci.

[B59] Duncan MK, Xie L, David LL, Robinson ML, Taube JR, Cui W, Reneker LW (2004). Ectopic Pax6 expression disturbs lens fiber cell differentiation. Invest Ophthalmol Vis Sci.

[B60] Pratt T, Price DJ, Erzurumlu R, Guido W and Molnar Z (2006). Dual roles of transcription factors in forebrain morphogenesis and development of axonal pathways.. Development and Plasticity in Sensory Thalamus and Cortex.

[B61] Dickson BJ (2002). Molecular mechanisms of axon guidance. Science.

[B62] Herrera E, Brown L, Aruga J, Rachel RA, Dolen G, Mikoshiba K, Brown S, Mason CA (2003). Zic2 patterns binocular vision by specifying the uncrossed retinal projection. Cell.

[B63] Plump AS, Erskine L, Sabatier C, Brose K, Epstein CJ, Goodman CS, Mason CA, Tessier-Lavigne M (2002). Slit1 and Slit2 cooperate to prevent premature midline crossing of retinal axons in the mouse visual system. Neuron.

[B64] Williams SE, Mann F, Erskine L, Sakurai T, Wei S, Rossi DJ, Gale NW, Holt CE, Mason CA, Henkemeyer M (2003). Ephrin-B2 and EphB1 mediate retinal axon divergence at the optic chiasm. Neuron.

[B65] Pratt T, Conway CD, Tian NM, Price DJ, Mason JO (2006). Heparan sulphation patterns generated by specific heparan sulfotransferase enzymes direct distinct aspects of retinal axon guidance at the optic chiasm. J Neurosci.

[B66] Pak W, Hindges R, Lim YS, Pfaff SL, O'Leary DD (2004). Magnitude of binocular vision controlled by islet-2 repression of a genetic program that specifies laterality of retinal axon pathfinding. Cell.

[B67] Drager UC, Olsen JF (1980). Origins of crossed and uncrossed retinal projections in pigmented and albino mice. J Comp Neurol.

[B68] Pratt T, Tian NM, Simpson TI, Mason JO, Price DJ (2004). The winged helix transcription factor Foxg1 facilitates retinal ganglion cell axon crossing of the ventral midline in the mouse. Development.

[B69] Herrera E, Marcus R, Li S, Williams SE, Erskine L, Lai E, Mason C (2004). Foxd1 is required for proper formation of the optic chiasm. Development.

[B70] Ziman M, Rodger J, Lukehurst S, Hancock D, Dunlop S, Beazley L (2003). A dorso-ventral gradient of Pax6 in the developing retina suggests a role in topographic map formation. Brain Res Dev Brain Res.

[B71] Quina LA, Pak W, Lanier J, Banwait P, Gratwick K, Liu Y, Velasquez T, O'Leary DD, Goulding M, Turner EE (2005). Brn3a-expressing retinal ganglion cells project specifically to thalamocortical and collicular visual pathways. J Neurosci.

[B72] Marcus RC, Mason CA (1995). The first retinal axon growth in the mouse optic chiasm: axon patterning and the cellular environment. J Neurosci.

[B73] Wang SW, Gan L, Martin SE, Klein WH (2000). Abnormal polarization and axon outgrowth in retinal ganglion cells lacking the POU-domain transcription factor Brn-3b. Mol Cell Neurosci.

[B74] Baumer N, Marquardt T, Stoykova A, Spieler D, Treichel D, Ashery-Padan R, Gruss P (2003). Retinal pigmented epithelium determination requires the redundant activities of Pax2 and Pax6. Development.

[B75] Mu X, Fu X, Sun H, Liang S, Maeda H, Frishman LJ, Klein WH (2005). Ganglion cells are required for normal progenitor- cell proliferation but not cell-fate determination or patterning in the developing mouse retina. Curr Biol.

[B76] Hogan BL, Horsburgh G, Cohen J, Hetherington CM, Fisher G, Lyon MF (1986). Small eyes (Sey): a homozygous lethal mutation on chromosome 2 which affects the differentiation of both lens and nasal placodes in the mouse. J Embryol Exp Morphol.

[B77] Baulmann DC, Ohlmann A, Flugel-Koch C, Goswami S, Cvekl A, Tamm ER (2002). Pax6 heterozygous eyes show defects in chamber angle differentiation that are associated with a wide spectrum of other anterior eye segment abnormalities. Mech Dev.

[B78] Davis J, Duncan MK, Robison WG, Piatigorsky J (2003). Requirement for Pax6 in corneal morphogenesis: a role in adhesion. J Cell Sci.

[B79] Ramaesh T, Collinson JM, Ramaesh K, Kaufman MH, West JD, Dhillon B (2003). Corneal abnormalities in Pax6+/- small eye mice mimic human aniridia-related keratopathy. Invest Ophthalmol Vis Sci.

[B80] Zaki PA, Collinson JM, Toraiwa J, Simpson TI, Price DJ, Quinn JC (2006). Penetrance of eye defects in mice heterozygous for mutation of Gli3 is enhanced by heterozygous mutation of Pax6. BMC Dev Biol.

[B81] Matsunami H, Takeichi M (1995). Fetal brain subdivisions defined by R- and E-cadherin expressions: evidence for the role of cadherin activity in region-specific, cell-cell adhesion. Dev Biol.

[B82] Barnett MW, Old RW, Jones EA (1998). Neural induction and patterning by fibroblast growth factor, notochord and somite tissue in Xenopus. Dev Growth Differ.

